# Differential requirement of Formyl Peptide Receptor 1 in macrophages and neutrophils in the host defense against *Mycobacterium tuberculosis* Infection

**DOI:** 10.21203/rs.3.rs-4421561/v1

**Published:** 2024-05-29

**Authors:** Tanvir Noor Nafiz, Poornima Sankar, Lokesh K Mishra, Robert P. Rousseau, Mohd Saqib, Selvakumar Subbian, Suraj P. Parihar, Bibhuti B. Mishra

**Affiliations:** 1 Department of Immunology and Microbial Disease, Albany Medical College, Albany, NY, USA.; 2 Public Health Research Institute, New Jersey Medical School, Rutgers University, Newark, NJ, USA; 3 Center for Infectious Diseases Research in Africa (CIDRI-Africa) and Institute of Infectious Diseases and Molecular Medicine (IDM), Division of Medical Microbiology, Faculty of Health Sciences, University of Cape Town, Anzio Road, Observatory 7925, Cape Town, South Africa.

**Keywords:** Formyl peptide receptors, *Mycobacterium tuberculosis*, Host defense, G-protein coupled receptors, Immunity

## Abstract

Formyl peptide receptors (FPR), part of the G-protein coupled receptor superfamily, are pivotal in directing phagocyte migration towards chemotactic signals from bacteria and host tissues. Although their roles in acute bacterial infections are well-documented, their involvement in immunity against tuberculosis (TB) remains unexplored. This study investigates the functions of Fpr1 and Fpr2 in defense against *Mycobacterium tuberculosis* (Mtb), the causative agent of TB. Elevated levels of Fpr1 and Fpr2 were found in the lungs of mice, rabbits and peripheral blood of humans infected with Mtb, suggesting a crucial role in the immune response. The effects of Fpr1 and Fpr2 deletion on bacterial load, lung damage, and cellular inflammation were assessed using a TB model of hypervirulent strain of Mtb from the W-Beijing lineage. While *Fpr2* deletion showed no impact on disease outcome, *Fpr1*-deficient mice demonstrated improved bacterial control, especially by macrophages. Bone marrow-derived macrophages from these *Fpr1*^*−/−*^ mice exhibited an enhanced ability to contain bacterial growth over time. Contrarily, treating genetically susceptible mice with Fpr1-specific inhibitors caused impaired early bacterial control, corresponding with increased bacterial persistence in necrotic neutrophils. Furthermore, ex vivo assays revealed that *Fpr1*^*−/−*^ neutrophils were unable to restrain Mtb growth, indicating a differential function of Fpr1 among myeloid cells. These findings highlight the distinct and complex roles of Fpr1 in myeloid cell-mediated immunity against Mtb infection, underscoring the need for further research into these mechanisms for a better understanding of TB immunity.

## Introduction

Tuberculosis (TB), an infectious disease caused by *Mycobacterium tuberculosis* (Mtb), continues to pose a substantial global health challenge. In 2022, TB was responsible for an estimated 10.6 million new cases and 1.3 million deaths. The impact of the disease has been further exacerbated by the COVID-19 pandemic^1^. While most individuals infected with Mtb can successfully eliminate the infection, a subset develops an asymptomatic latent Mtb infection (LTBI). Notably, about 5–10% of those with LTBI progress to active tuberculosis (ATB) over their lifetime^2,3^. Upon infection, *Mycobacterium tuberculosis* (Mtb) is promptly internalized by nonspecific phagocytic cells within the pulmonary system, which serve to contain and manage the bacterial burden. Although the T cell-mediated immune response plays a pivotal role in controlling Mtb, it characteristically necessitates a period of 2–3 weeks in mice and 4–6 weeks to establish an Mtb-specific T cell response in humans. This delay highlights the critical window during which the initial innate immune mechanisms are essential for the initial containment of the infection. Therefore, the early immune response, mediated by innate cells such as neutrophils and macrophages, is critical for host defense against Mtb infection. Sensing of Mtb and Mtb-derived products plays a central role in the innate immune response against TB. This pathogen recognition mechanism is mediated by pattern recognition receptors (PRRs) essential for the initial detection of Mtb. Notably, toll-like receptors (TLRs), nucleotide-binding oligomerization domain (NOD)-like receptors (NLRs), C-type lectin receptors (CLRs), complement receptors (CRs), scavenger receptors (SRs), absent in melanoma 2 (AIM2)^4^, aryl hydrocarbon receptor (AhR)^5^, and CD14 receptors^6^ have been identified as key PRRs in recognizing Mtb pathogen-associated molecular patterns (PAMPs). In addition to these receptors, formyl peptide receptors (FPRs) are atypical PRRs that play a pivotal role in the host’s defense against a broad spectrum of infections and inflammatory responses^7^.

Formyl peptide receptors (FPRs) are a class of G protein-coupled receptors integral to host defense mechanisms and inflammatory responses^8,9^. To date, eight murine and three human isoforms: FPR1, FPR2, and FPR3 of FPRs have been identified. These receptors exhibit unique roles in immune modulation, distinguished by their expression patterns and ligand specificities^10^. FPR1 is primarily recognized for its function in directing neutrophil chemotaxis towards short (3–5 amino acids) N-formylated peptides, which are typically products of bacterial metabolism or released by mitochondria following cellular damage, thereby triggering an immune response^10^. FPR1 is the phagocyte receptor for plague pathogen, *Yersinia pestis*^11^. Bacterial infections caused by *E.coli*^12^, *Listeria monocytogenes*^13^, *Streptococcus pneumoniae*^14^, and methicillin-resistant *Staphylococcus aureus*^15^, are sensed by FPR1 that play a critical role in the host defense against these pathogens. Studies indicate that FPR1 not only responds to these bacterial peptides but also orchestrates several neutrophil activities, including degranulation and superoxide production, and is vital for effective bacterial eradication^16^. Moreover, a recent report has implicated the regulatory role of FPR1 in protecting hosts from bleomycin-induced pulmonary fibrosis where neutrophil-specific FPR1 plays a role in scar formation^17^. These studies highlight the antimicrobial and pro-inflammatory role of FPR1 during infections and inflammation. FPR2, though structurally similar to FPR1, interacts with a wider array of ligands, including longer N-formylated peptides from pathogens such as *Staphylococcus aureus* and *Listeria monocytogenes*^13^. Additionally, FPR2 binds to host-derived molecules like lipoxins and serum amyloid A, which play roles in inflammation resolution and immune response modulation^18,19^. This suggests a dual role for FPR2 in both promoting and mitigating inflammatory processes. Interestingly, lack of FPR1 and 2 causes severe inflammation and bacterial burden in a pneumococcal meningitis model, suggesting the non-redundant role of these FPRs in host defense^14^. FPR3 is the least explored among the isoforms and exhibits selective expression mainly in myeloid cells excluding neutrophils. The specific functions and ligands of FPR3 are not well-documented; however, it is hypothesized to influence immune cell migration and possibly engage in non-inflammatory roles within the immune system^20^. Collectively, the distinct yet overlapping functionalities of the FPR isoforms across different immune cells underscore their potential as intriguing targets for therapeutic development, offering promising avenues for enhancing immune response precision and efficacy.

Formylated peptides are characteristic PAMPs of bacterial pathogens released in the host milieu as a consequence of microbicidal activities of immune cells or during mitochondrial protein synthesis^21^. Mycobacterial formylated peptides are potentially released during bacterial lysis, which are potential ligands for FPRs expressed on neutrophils and monocytes/macrophages^22^. This interaction allows mycobacteria to activate atypical FPRs, facilitating an immune response. Studies have highlighted that blood monocytes can be activated by these peptides, further underscoring their significance in immune signaling pathways. FPR1, in particular, can be activated by *Mycobacterium butyricum*, an attenuated strain of the bacterium, highlighting a specific pathogen-host interaction^23^. Moreover, increased expression of FPR1 on monocytes has been associated with active TB^24^, suggesting its potential as a biomarker for disease activity. Recent studies have demonstrated elevated expression of FPR1 in TB lesions, with the FPR1-specific pentapeptide cFLFLF accumulating in lung granulomas in mice and non-human primates^25^. A study using an *in vitro* human granuloma model showed that polyethylene glycol modified (PEGylated) cFLFLF binds to neutrophils and macrophages within granulomas^26^, suggesting a role for FPR1 in the phagocytic response to Mtb. Despite these advances, the specific functions of FPR1 and FPR2 in host defense against TB remain poorly understood.

In this study, we aimed to delineate the roles of FPR1 and FPR2 in various models of TB resistance and susceptibility. We focused on assessing how genetic deletion or pharmacological blockade of FPR1 affects the antimicrobial functions of neutrophils and macrophages. Our research demonstrates that while FPR2 does not significantly influence TB resistance, FPR1 plays a variable role in modulating the host response, showing different impacts in resistant versus susceptible host backgrounds. Furthermore, FPR1 is found to have distinct roles in neutrophils and macrophages, contributing differently to the host’s defense mechanisms against TB. Our study brings to light a previously underappreciated facet of these atypical PRRs in mediating TB immunity, underscoring the potential of FPR1 and FPR2 as critical targets for modulating the immune response in TB. This nuanced understanding of FPR1 and FPR2 could guide targeted therapeutic strategies aimed at enhancing TB resistance.

## Results

### FPR expression is induced by Mtb infection

**Figure 1.**
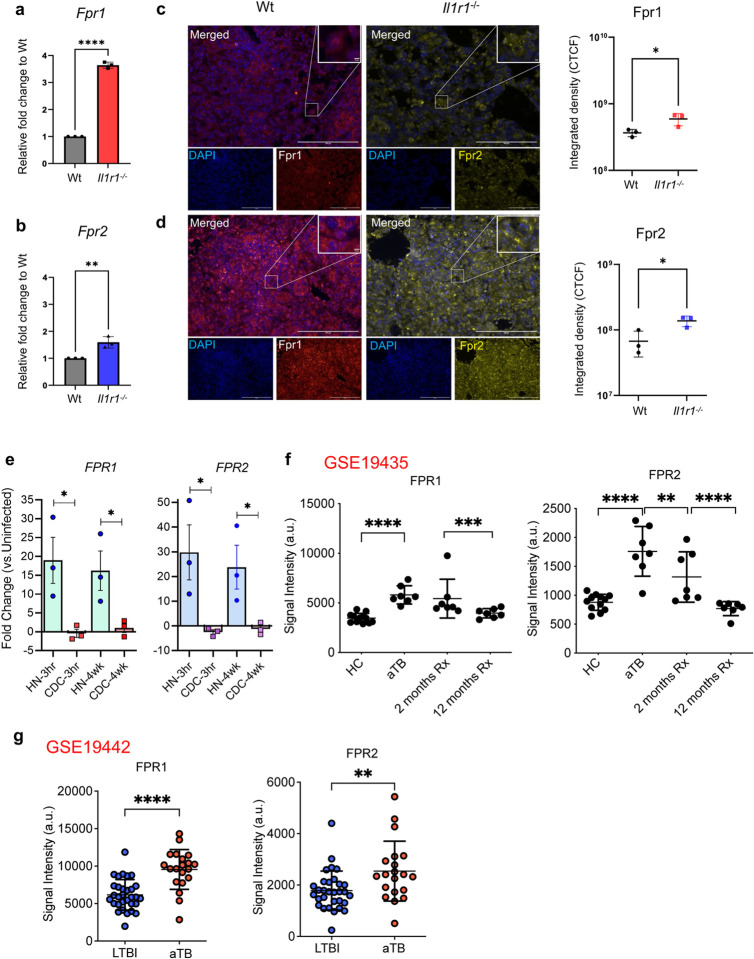
Fpr1 and 2 expression in the lungs is associated with TB disease. (**a**) Wild type (Wt) and *Il1r1*^*−/−*^ mice were infected via aerosol with Mtb HN878 smyc’:: mCherry (Mtb), delivering approximately 100 CFU to the lungs. At 26 dpi, Fpr1 (**a**) and Fpr2 (**b**) expressions were quantified in the lungs using qPCR. Expression levels in *Il1r1*^*−/−*^ mice were calculated relative to those in Wt C57BL/6 (Wt) mice. (**c, d**) Representative immunofluorescence images of formalin-fixed paraffin-embedded (FFPE) lung sections from Mtb-infected Wt and *Il1r1*^*−/−*^ mice. Left panels: DAPI (blue) stains nuclei, Fpr1 (red). Right panels: DAPI (blue), Fpr2 (yellow). Quantification of Fpr1 and Fpr2 expressions in lung sections expressed as corrected total fluorescence intensity (CTCF) from three fields of view per group, representing one of two experiments. Data represent n=3 samples per time point. Error bars show Mean ± SEM. Statistical analysis was performed using an unpaired t-test. *P<0.05, ****P<0.00001. **(e)** qPCR analysis of FPR1 and FPR2 expressions in rabbit lungs post Mtb infection with strains HN878 and CDC1551 at 3 hours and 4 weeks. **(f)** Formyl Peptide Receptor (FPR) 1 and 2 expression in human TB before and after anti-TB therapy: RNA-seq data was extracted from publicly available dataset previously published (GSE19435). Data is comprised of whole blood transcriptional signatures obtained from two different cohorts. Data sets were downloaded from NCBI as the Longitudinal TB Treatment in a UK cohort (GSE19435) and (**g**) whole blood transcriptional signatures in latent TB (LTBI) and active TB in a South African Cohort (GSE19442). Genes were identified based on their Ilumina IDs; FPR1(ILMN_2092118), FPR2 (ILMN_2392569, ILMN_1740875), extracted, plotted and analysed by unpaired student t-test versus the indicated groups. **p<0.01, ***p<0.001 and ****p<0.0001.

To examine the dynamics of Fpr1 expression during Mtb infection, we used two mouse models: the relatively resistant C57BL/6 mice (designated as Wt) and the susceptible *Il1r1*^*−/−*^ mice (on a C57BL/6 background)^27,28^. Both groups were infected with the hypervirulent Mtb strain HN878, which belongs to the W-Beijing lineage 2 strains known to recapitulate pathologies similar to those observed in human TB^29,30^. We assessed the expression of Fpr1 and Fpr2 in the lungs of these animals. Elevated expression levels of Fprs were observed at the mRNA level in *Il1r1*^*−/−*^ mice at 25 days post-infection (dpi), compared to wild type (Wt) mouse lungs, (Fig. 1a, b). In alignment with the mRNA data, immunofluorescence staining of lung sections revealed higher levels of Fpr1 and Fpr2 proteins in the lungs of *Il1r1*^*−/−*^ mice than their Wt counterparts (Fig. 1c, d). These findings suggest that Mtb infection markedly upregulates Fpr expression and Fpr levels in genetically susceptible mice may be associated with increased vulnerability to Mtb infection.

Next, we utilized a rabbit model of TB by infecting outbred rabbits with either Mtb strain CDC1551, which typically induces latent infection, or the more virulent strain HN878, associated with caseating/necrotic TB^31,32^. Analysis of FPR expression in the lungs demonstrated that rabbits infected with the HN878 strain showed significantly increased expression of FPR1 and FPR2. Notably, elevated levels of these receptors persisted at 4 weeks post-infection compared to those infected with the CDC1551 strain (Fig. 1e). These results, along with findings from susceptible *Il1r1*^*−/−*^ mice, suggest that heightened FPR expression in response to hypervirulent HN878 strain infection may be crucial in the pathogenesis of TB.

Furthermore, we investigated whether FPR1 and FPR2 expression is altered in human TB by analyzing publicly available transcriptome databases. Specifically, we examined a cohort from the United Kingdom (GSE19435) that was longitudinally monitored for 12 months following antibiotic treatment^33^. We observed that expression levels of FPR1 and FPR2 in peripheral blood cells were elevated in patients with active pulmonary TB compared to healthy controls (HCs). Notably, these expression levels returned to those comparable to HCs after 12 months of successful anti-TB treatment (Fig. 1f), indicating that FPR expression is linked to active disease and depends on the Mtb antigenic load. In parallel, we reanalyzed transcriptome datasets from an independent cohort in South Africa (GSE19442), comparing individuals with latent TB infection to patients with active sputum smear-positive TB. This analysis revealed that patients with active TB expressed higher levels of FPR1 and FPR2 compared to those with latent infection, suggesting that these receptors are induced by Mtb infection, and their expression correlates with symptomatic disease (Fig. 1g). Taken together, our findings across mouse, rabbit, and human models demonstrate that FPR1 and FPR2 expressions are associated with disease severity and may play a significant role in the pathogenesis of TB.

### Fpr1 deletion improved TB outcomes in BL/6 mice

**Figure 2.**
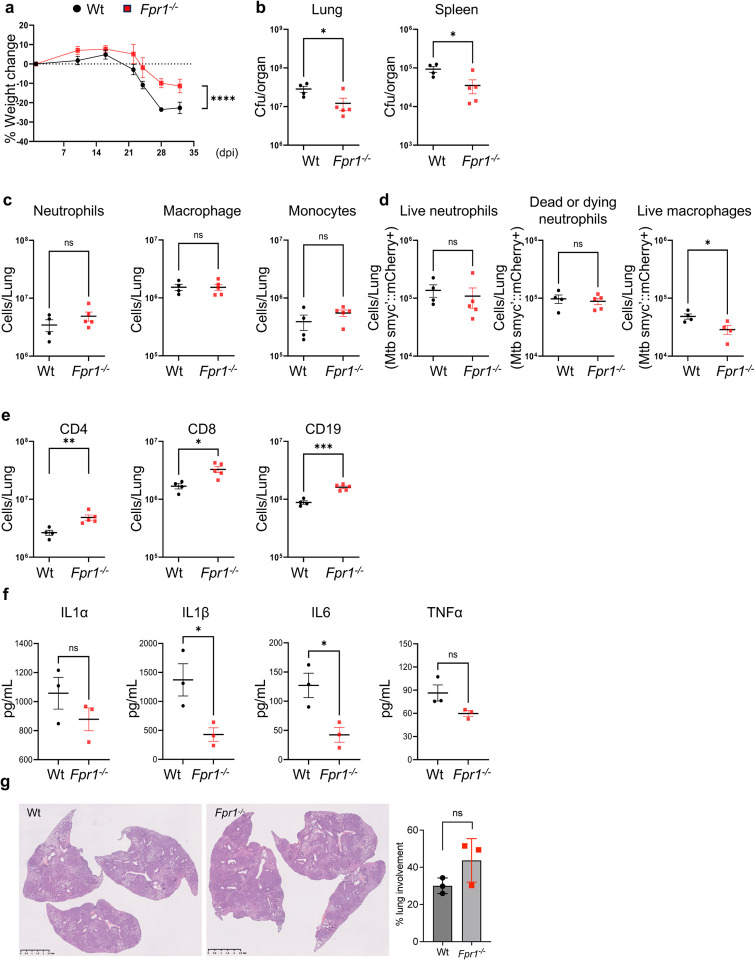
Protective effects of Fpr1 deletion on tuberculosis outcomes in mice. Wt and *Fpr1*^*−/−*^ mice on a C57BL/6 background were aerosol-infected with Mtb HN878 reporter bacteria as per the protocol in Figure 1. Necropsies and tissue analyses were conducted at 32 dpi, with 4–6 mice per group. **(a)** Percentage of weight change upto 32 dpi in Mtb-infected Wt and *Fpr1*^*−/−*^ mice. **(b)** Bacterial load in the lungs and spleens measured in CFU per ml at 32 dpi in both Wt and *Fpr1*^*−/−*^ mice. **(c)** Flow cytometry assessment of myeloid cells, including neutrophils, macrophages, and monocytes at 32 dpi in both Wt and *Fpr1*^*−/−*^ mice. **(d)** Flow cytometry assessment of infected neutrophils and macrophages at 32 dpi in both Wt and *Fpr1*^*−/−*^ mice. Live infected neutrophils were marked with viability dye-CD11b+Ly6G+smyc’::mCherry+, while dead or dying neutrophils were identified using viability dye+CD11b+Ly6G+smyc’::mCherry+. Live infected macrophages were marked with viability dye-CD11b+Ly6G-CD11c+MHCII+SiglecF-smyc’::mCherry+. **(e)** Flow cytometry for T-lymphocytes (CD4+, CD8+) and B lymphocytes (CD19+) at 32 dpi in Wt and *Fpr1*^*−/−*^ mice. **(f)** Quantification of cytokines (IL-1α, IL-1β, IL-6, TNF-α) in lung homogenates from both Wt and *Fpr1*^*−/−*^ mice at 35 dpi. n=3 per group. **(g)** Lung Histopathology: Images and blind scoring of inflammatory lesions in the lungs of Wt and *Fpr1*^*−/−*^ mice at 35 dpi. n=4–5 mice per group. Error bars represent Mean ± SEM. Statistical analysis involved a two-way ANOVA for panel (a), with significance determined by Tukey’s multiple comparison test (****p<0.0001). Unpaired t-tests were conducted for panels (**b-g**). *P<0.05, ***p<0.001, and “ns” denotes non-significant results.

Given the observed inductions in FPR1 and FPR2 expression during Mtb infection and its potential association with host susceptibility, it is critical to understand the specific roles that FPR1 and FPR2 might play in immune mechanisms of protection or pathology during TB. To address this, we infected Wt, *Fpr1*^*−/−*^ and *Fpr2*^*−/−*^ mice, all in the C57BL/6 background and have been previously reported^12^ with Mtb HN878 smyc’::mCherry bacteria, which serve as a tool for monitoring of bacterial infection and survival in various host cells. After 32 dpi, we measured weight loss, bacterial burden in the lung and spleen, cellular infiltration to the lung and histopathology of the lung, as measures of infection outcomes. Compared to the Wt mice, *Fpr1*^−*/*−^ mice significantly lost less body weight and had lower bacterial load measured as colony forming unit (CFU) counts in the lung and spleen. Intriguingly, Fpr2 deletion (*Fpr2*^*−/−*^) did not impact weight loss or bacterial growth as infection in these animals led to comparable body weight and CFUs in the lung as Wt (Fig. 2a, b; **Supplementary Fig. 2a**). These observations in weight change and bacterial burden indicated a protective effect of Fpr1 deletion on infection outcomes.

Next, we determined the impact of Fpr deletion on immune cell dynamics in the lung following Mtb infection at 32 dpi. The deletion of *Fpr1* or *Fpr2* had no significant impact on overall leukocyte infiltration as the absolute number of neutrophils, macrophages, and monocytes were comparable in Wt and *Fpr1*^*−/−*^ and *Fpr2*^*−/−*^ mice (Fig. 2c; **Supplementary Fig. 2b**) suggesting that Fprs may not regulate immune cell trafficking during Mtb infection. However, *Fpr1* deletion led to a significant reduction in Mtb-infected macrophages, harboring Mtb smyc’::mCherry (Fig. 2d), though the number of infected neutrophils (both live and dead) were not affected (**Supplementary Fig. 2c)**, indicating a potential inhibitory effect of Fpr1 on macrophage’s ability to control Mtb in the lung microenvironment of the hosts known to exhibit relatively better resistance to TB disease.

Notably, the deletion of *Fpr1* also appeared to increase the number of CD4, CD8-T cells and CD19^+^ B-cells in the lung whereas the lymphocyte numbers were not affected by *Fpr2* deletion. The increase in number of lymphocytes in *Fpr1*^*−/−*^ animals was associated with an overall decline in the bacterial burden, consistent with the protective role of these cells in TB immunity (Fig. 2e; **Supplementary Fig. 2d**). Moreover, *Fpr1* deletion led to a reduction in pro-inflammatory cytokines, IL-1b and IL-6 in the lungs compared to Wt mice (Fig. 2f). Neither Fpr1 nor Fpr2 deletion had any impact on the lung pathology compared to wt mice lungs (Fig. 2g; **Supplementary Fig. 2e**). These findings highlight the potential of Fpr1 as a modulator of not only the innate antimicrobial response but also of T cell infiltration in the lungs during Mtb infection. Overall, the data collectively suggest that Fpr1 plays multifaceted roles in the immune response to TB, influencing various aspects of both innate and adaptive immunity. However, no apparent effect on neutrophil response to Mtb infection, whether antimicrobial or in terms of trafficking, was observed upon Fpr1 deletion, likely due to the C57BL/6 genetic background of these hosts.

### Blockade of Fpr1 in *Il1r1*-deficient mice impaired bacterial control affecting neutrophils

**Figure 3.**
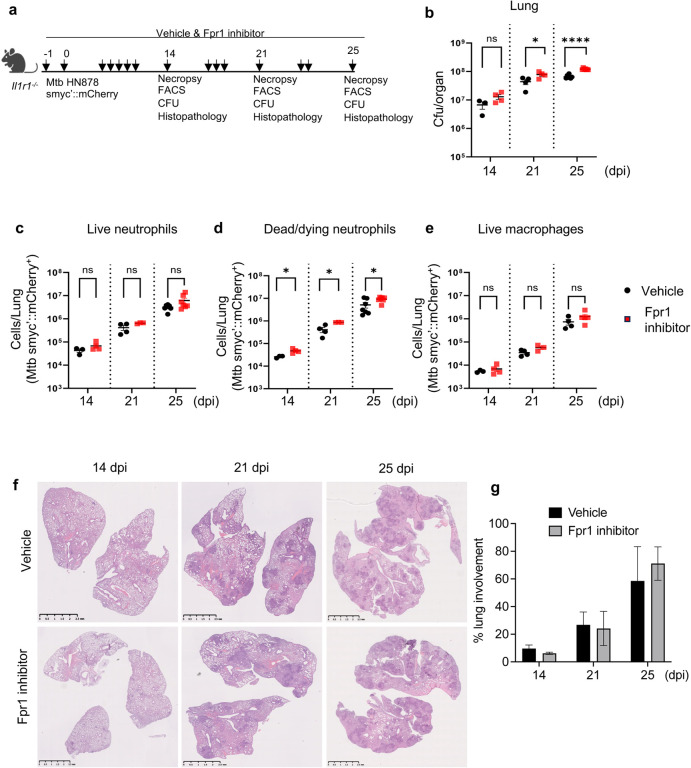
Blocking Fpr1 in susceptible mice increased bacterial growth in the lungs. **(a)** Experimental setup: *Il1r1*^*−/−*^ mice were exposed to an aerosol containing approximately 100 CFU of Mtb HN878 reporter bacteria. Starting one day before infection (day −1), the mice were given either a vehicle or Fpr1 inhibitors every other day. The Fpr1 inhibitors used were a combination of Cyclosporin H (4 mg/kg) and HCH6–1 (4 mg/kg), administered orally. Measurements were taken on days 14, 21, and 25 post-infection. **(b)** Bacterial load in the lungs was determined by CFU counts. **(c)** Flow cytometry was used to assess Mtb-infected neutrophils (live on the left), (**d**) dead/dying on the middle and (**e**) macrophages in the right. **(f)** Lung Histopathology: Representative images of H&E-stained FFPE lung sections are shown for the specified infection times. **(g)** Quantification of necrotic lesion areas illustrates the progression of disease over time in both vehicle-treated and inhibitor-treated mouse lungs. Data are from n=3–7 mice per group. Results from day 25 are combined from two separate experiments. Error bars represent the mean ± SEM. Statistical significance was assessed using an unpaired t-test compared to respective controls. *p<0.05; ****p<0.0001; ‘ns’ denotes non-significant results.

Given the elevated expression of Fpr1 in the lungs of Mtb-infected *Il1r1*^*−/−*^ mice, coupled with observations that Fpr1 deletion in Wt C57BL/6 mice enhances bacterial control and improves outcomes, we examined the effect of Fpr1 blockade on disease progression in the genetically susceptible *Il1r1*^*−/−*^ model. We employed Fpr1 inhibitors, specifically Cyclosporin H and HCH6–1, to assess their impact. Cyclosporin H, a well-known FPR1 antagonist, retains the receptor in an inactive state^34^, while HCH6–1 inhibits downstream signaling of Fpr1^35^. Following infection of *Il1r1*^*−/−*^ mice with Mtb HN878 smyc’::mCherry, the inhibitors were administered orally every other day, as depicted in schematics (Fig. 3a). Necropsy at 14-, 21-, and 25 dpi allowed for the assessment of bacterial burden, immune cell infiltration, and histopathology to gauge disease outcomes. The time point of 25 dpi was selected because these mice typically succumb to infection by 28 dpi. Fpr1 inhibition led to a significant increase in bacterial CFU at 21 and 25 dpi (Fig. 3b). Flow cytometry analysis showed that Fpr1 inhibition did not alter the overall infiltration of leukocytes and lymphocytes (**Supplementary Figure 3a, b**). Although the presence of bacteria-containing neutrophils and macrophages was comparable between the vehicle-treated and Fpr1-inhibited *Il1r1*^*−/−*^ mice, a significantly greater abundance of dead/dying neutrophils harboring Mtb smyc’::mCherry was observed in the Fpr1-inhibited group, indicating a detrimental impact on neutrophils likely due to inadequate bacterial control (Fig. 3c–e). Further, lung histology assessments corroborated these findings, depicting worsened conditions in Fpr1-inhibited mice (Fig. 3f, g). Collectively, our results highlight the critical role of Fpr1 in mediating early control of Mtb infection, predominantly through neutrophils, and suggest that inhibition of this receptor in a susceptible genetic background predisposes the host to exacerbated disease outcomes plausibly by regulating neutrophil antibacterial functions.

### Protective role of Fpr1 in the susceptible C3HeB mice

**Figure 4.**
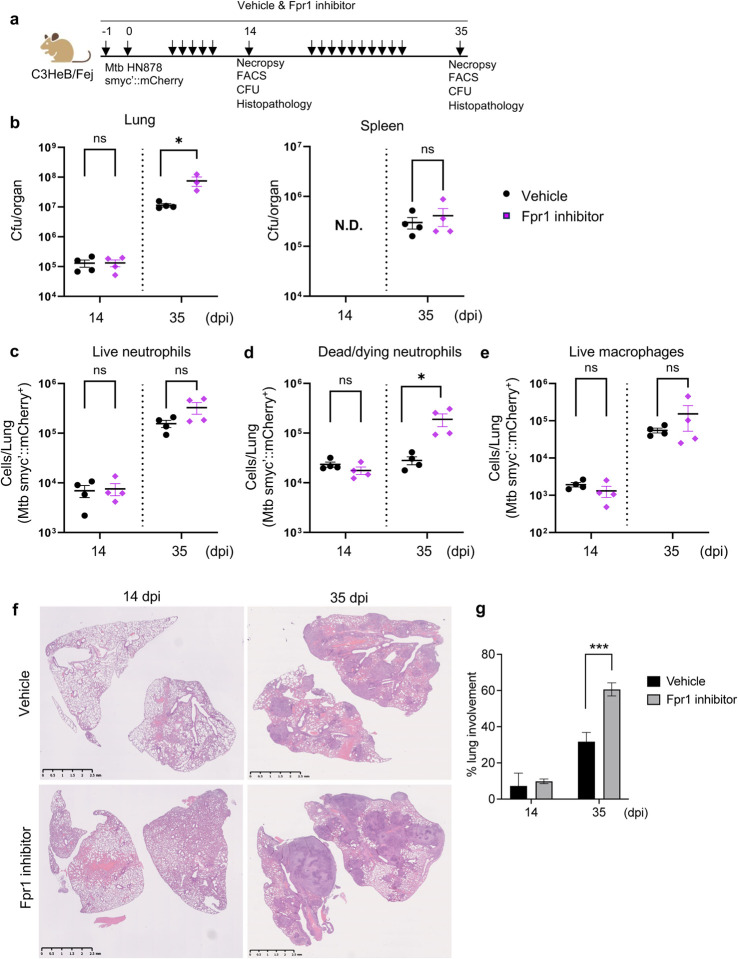
Fpr1 blockade impairs bacterial control in immunocompetent C3HeB mice. (**a**) Experimental design: C3HeB mice were infected with Mtb HN878 reporter bacteria via aerosol and treated with either a vehicle or Fpr1 inhibitors according to the schematic. Evaluations were conducted at 14- and 35 dpi. **(b)** Bacterial burden in the lungs and spleens of both vehicle-treated and inhibitor-treated mice was measured and expressed as colony-forming units (CFU). **(c)** Flow cytometry was used to assess Mtb-infected neutrophils (live on the left); (**d**) dead/dying on the middle) and (**e**) macrophages (right panel) at the specified time points post-infection. (**f**) Representative histopathology images of H&E-stained FFPE lung sections. (**g**) Quantification of necrotic lesion areas in the lungs at 14 and 35 dpi for both vehicle- and inhibitor-treated mice. n = 4 per group. Error bars represent Mean ± SEM. Statistical significance was assessed using an unpaired t-test compared to respective controls. *p<0.05; ***p<0.001; ‘ns’ denotes a non-significant result.

The unexpected phenotype in *Il1r1*^*−/−*^ mice following Fpr1 inhibition prompted an investigation into whether Fpr1’s protective functions during early Mtb infection are mediated by neutrophils. We utilized another susceptible mouse strain, C3HeB, known for its neutrophil-mediated TB pathogenesis^36,37^ and development of a range of TB lesions that recapitulate the pathological features of human TB^38,39^. This model provided a potentially translatable insight into Fpr1’s role in TB susceptibility. Immunofluorescence staining of Fpr1 in C3HeB mice demonstrated a specific induction at 35 dpi, suggesting a correlation between Fpr1 expression and TB susceptibility (**Supplementary Fig. 4a, b**). To further elucidate Fpr1’s role, we administered the same pharmacological inhibitors used previously (Fig. 4a). At 14 dpi, no significant difference in bacterial burdens was observed in the lungs between the control and Fpr1-inhibited groups; however, a significant increase in CFU was observed in the lungs but not in the spleen at 35 dpi (Fig. 4b). Flow cytometry analysis at this early stage also showed no differences in the counts of live and dead neutrophils harboring Mtb smyc’::mCherry, macrophages, and corresponding CFUs. Notably, as the infection progressed to 35 dpi, Fpr1-inhibited mice displayed a significant increase in dead/dying neutrophils harboring Mtb (Fig. 4c–e), which corresponded with an elevated overall bacterial load in the lung (Fig. 4b, **left panel**). No significant changes in the overall abundance of neutrophils, macrophages, monocytes, T- and B-lymphocytes were observed in the Fpr1-inhibited lungs compared to the vehicle-treated lungs (**Supplementary Fig. 5a, b**). While Fpr1 did not affect the bacterial clearance capabilities of live neutrophils and macrophages, the increased number of infected dead/dying neutrophils suggests a defect in bacterial containment, potentially leading to necrotic cell death driven by bacterial virulence^40^. Furthermore, the elevated CFU and abundance of dead/dying neutrophils with Mtb smyc’::mCherry at 35 dpi were associated with extensive tissue necrosis, as evidenced by histopathology analysis (Fig. 4f, g). These findings underscore a potentially unique aspect of Fpr1’s role in host defense, particularly in more susceptible models, where its absence significantly impairs the bacterial control capacity of neutrophils that are the predominant myeloid cells in the Mtb-infected lungs.

### Differential roles of Fpr1 in neutrophil and macrophage responses to Mtb infection.

**Figure 5.**
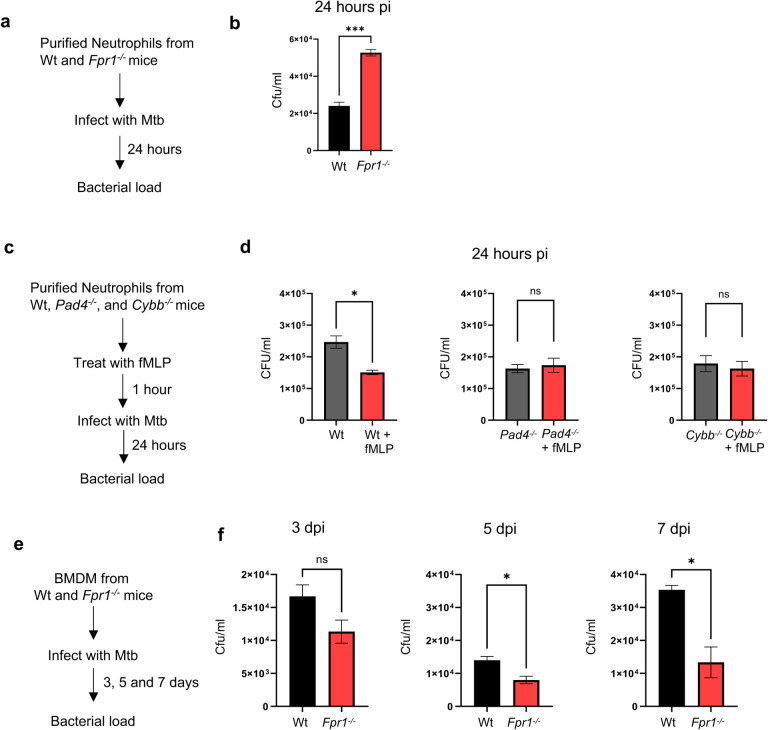
Fpr1 plays a contrasting role in macrophages and neutrophils during Mtb infection. **(a)** Experimental setup: Bone marrow-derived neutrophils from Wt and *Fpr1*^*−/−*^ mice were isolated using magnetic cell sorting (MACS) and infected with Mtb HN878 at a multiplicity of infection (MOI) of 3.0 for 4 hours. After removing extracellular bacteria through washing, cells were further incubated for 24 hours. The intracellular bacterial load was then assessed using CFU analysis. **(b)** Intracellular bacterial load in neutrophils is presented as CFU counts. **(c)** Experimental setup: Bone marrow-derived neutrophils from Wt, *Pad4*^*−/−*^, and *Cybb*^*−/−*^ mice were isolated using MACS and infected with Mtb HN878 at an MOI of 3.0, as described in (**a**), with or without the addition of 100nM fMLP. (**d**). Bacterial burden in these neutrophils was measured at 24 hours post-infection and is shown as CFU. **(e)** Experimental setup: Bone marrow-derived macrophages (BMDMs) from Wt and *Fpr1*^*−/−*^ mice were infected with Mtb HN878 at an MOI of 3.0. **(f)** Bacterial load in BMDMs was determined at various time points post-infection and expressed as CFU counts. The experiments were conducted with n=3 replicates per group and are representative of two independent experiments. Error bars represent Mean ± SEM. Statistical analysis was performed using unpaired t-tests. *p<0.05; ‘ns’ denotes a non-significant result.

To further investigate Fpr1’s function in TB pathogenesis, specifically its impact on neutrophils and macrophages in controlling bacterial infection, we first isolated neutrophils from both Wt and *Fpr1*^*−/−*^ mice (Fig. 5a). These cells were then infected with Mtb strain HN878, and the bacterial load within these cells was assessed 24 hours post-infection (hpi) using CFU assays. Neutrophils lacking Fpr1 exhibited a significantly higher bacterial burden compared to their Wt counterparts (Fig. 5b). This finding suggests that Fpr1 may play a critical role in controlling intracellular Mtb growth within the neutrophils. To further examine whether activating Fpr1 affects the antibacterial function of neutrophils, we pre-treated the neutrophils with an Fpr1 agonist, fmLP, before infecting them with Mtb. The intracellular bacterial burden was assessed 24hpi by CFU counting (Fig. 5c). Consistent with the results of the genetic deletion, neutrophils stimulated with fmLP showed enhanced efficacy in controlling intracellular bacterial growth, supporting the role of Fpr1 in regulating neutrophil’s antibacterial properties (Fig. 5d).

Neutrophil antibacterial responses are driven by several mechanisms^41,42^, including the formation of neutrophil extracellular traps (NETs)^43^ and the generation of reactive oxygen species (ROS)^44^. The enzyme peptidyl arginine deaminase 4 (Pad4) is essential for NET formation^45,46^ and plays a critical antibacterial role^47^. Similarly, the cytochrome b-245 beta subunit (Cybb) is a key component of the ROS-producing NADPH oxidase complex^48,49^. To explore the role of these microbicidal mechanisms during Mtb infection, we utilized neutrophils from *Pad4*^*−/−*^ or *Cybb*^*−/−*^ mice, which are deficient in NET and ROS production, respectively. These neutrophils were pretreated with the Fpr1 agonist fmLP, and intracellular Mtb growth was measured as previously described. Remarkably, both *Pad4*^*−/−*^ or *Cybb*^*−/−*^ neutrophils failed to control intracellular Mtb growth (Fig. 5c and 5d). These results suggest that Fpr1 activation enhances the antimycobacterial functions of neutrophils, which are dependent on both NET and ROS production.

**Figure 6:**
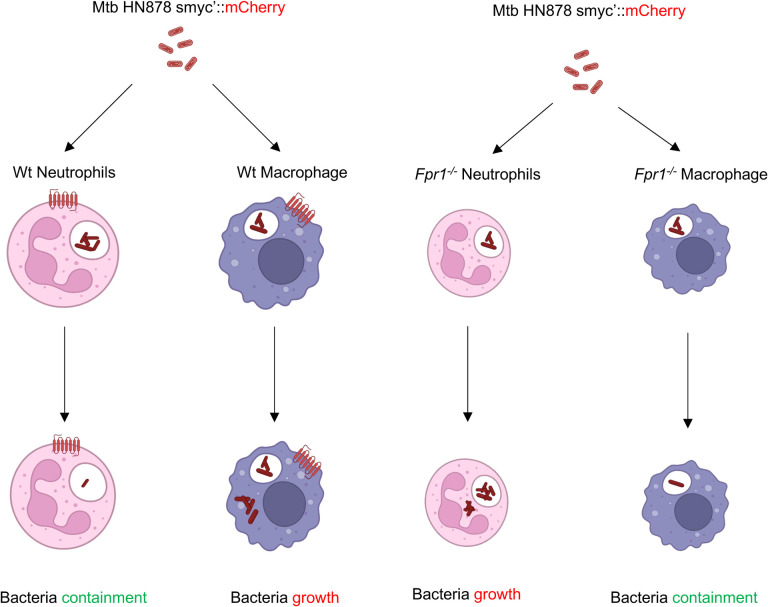
Model depicting the differential roles of Fpr1 in myeloid cell anti-mycobacterial functions. This model illustrates the contrasting effects of Fpr1 expression in neutrophils and macrophages during Mtb infection. Fpr1 expression in neutrophils is essential for effectively controlling Mtb growth within these cells. Conversely, in macrophages, Fpr1 expression impedes their ability to control intracellular Mtb growth, as evidenced by a reduced bacterial burden in macrophages lacking Fpr1. This model highlights the previously unrecognized importance of Fpr1 in the immune response of myeloid cells against tuberculosis. Graphic was designed by www.biorender.com.

In a parallel set of experiments, bone marrow-derived macrophages (BMDMs) were isolated from both Wt and *Fpr1*^*−/−*^ mice and subsequently infected with Mtb. Bacterial counts were assessed on 3, 5, and 7 dpi, by CFU counting. In contrast to the results seen with neutrophils, macrophages lacking Fpr1 showed an enhanced ability to clear the bacteria over time (Fig. 5e, f). These findings highlight a complex and seemingly opposing role of Fpr1 in the immune response dynamics of neutrophils and macrophages against Mtb infection. While Fpr1 appears to be crucial for neutrophils to effectively control intracellular bacterial growth, macrophages seem to perform better in bacterial clearance in its absence. This distinct functionality of Fpr1 in neutrophils versus macrophages may explain the phenotypes observed in susceptible and resistant hosts, where Mtb infection induces a neutrophil- and macrophage-dominated inflammatory lesions respectively in the lung^50,51^ (see model in Fig. 6).

## Discussion

In this study, we explored the role of formyl peptide receptor 1 (FPR1) in the immune response to Mtb infection, with a focus on its impact across different mouse models. Our findings highlight a complex, context-dependent role of FPR1 in modulating the host’s ability to manage Mtb infection, particularly in terms of bacterial clearance by neutrophils and macrophages. The differential roles of Fpr1 in neutrophils and macrophages became evident in different host backgrounds. In the C57BL/6 and *Fpr1*^*−/−*^ mice, we observed that Fpr1 deficiency led to increased bacterial proliferation in neutrophils, whereas macrophages from *Fpr1*^*−/−*^ mice showed enhanced bacterial containment capabilities. These results underscore a potentially dual role of Fpr1, where it is crucial for optimal neutrophil function, but limits the bactericidal efficiency of macrophages.

The use of pharmacological inhibitors Cyclosporin H and HCH6–1 provided critical insights into the functional dynamics of Fpr1 during Mtb infection. In the susceptible *Il1r1*^*−/−*^ mouse model, Fpr1 inhibition exacerbated disease progression, highlighting the importance of Fpr1 activity in controlling bacterial spread in this context. Notably, while leukocyte infiltration was unaffected by the inhibition, there was a significant increase in the number of dead neutrophils harboring bacteria. This suggests that the inhibition of Fpr1-dependent antimicrobial mechanisms in neutrophils may elevate the intracellular bacterial load within these cells, which in turn could induce cell death either through direct bacterial virulence or by influencing NETosis, a process known to participate in both bacterial killing and tissue damage. Further mechanistic studies are necessary to elucidate the detailed roles of Fprs in TB immunity, particularly how they influence neutrophil behavior and the overall outcome of the infection.

Our study in the C3HeB mouse model, which is notably susceptible to Mtb, further underscores the critical role of Fpr1 in modulating neutrophil functions. During the initial stages of infection, there were no discernible differences in bacterial burdens between the Fpr1-inhibited and control groups. However, as the infection progressed, the absence of Fpr1 significantly compromised bacterial control, particularly within dead/dying neutrophils. This pattern suggests a protective role for Fpr1 that becomes increasingly crucial over the course of the disease, especially in controlling bacterial loads within TB lesions where neutrophils are the predominant myeloid cells. These findings indicate that Fpr1 stimulation in neutrophils plays a pivotal role in controlling intracellular bacterial growth. Considering the effect of fmLP on neutrophils’ ability to control Mtb growth, Fpr1 agonists could potentially be developed as host-directed therapeutics for TB. Such compounds would need rigorous validation in preclinical models to evaluate their efficacy and safety in enhancing neutrophil-mediated bacterial clearance, offering a promising avenue for TB treatment strategies that target host immune responses.

In sum, our studies highlight Fpr1 as a crucial modulator in the host’s defense against TB, influencing both innate and adaptive immune responses. The role of Fpr1 in enhancing the bactericidal capacity of neutrophils identifies it as a valuable target for therapeutic strategies aimed at bolstering the host’s resistance to TB. Intriguingly, the observed opposing effect on macrophage antibacterial activity suggests a potential immune evasion strategy by Mtb. This bacterium might activate Fpr1 upon entry into the lungs, allowing it to evade destruction by these phagocytes. This proposed mechanism could facilitate the pathogen’s establishment, infection, and dissemination. Given these dynamics, future research should focus on delineating the specific signaling pathways and molecular mechanisms through which Fpr1 influences these distinct immune cell functions. Such studies are essential for developing targeted interventions that could enhance the effectiveness of TB treatment and management, potentially incorporating Fpr1 modulation as a strategic component in host-directed therapies.

## Materials and Methods

### Ethics statement

All animal procedures followed the standards set by the National Institutes of Health “Guide for the Care and Use of Laboratory Animals.” The Institutional Animal Care and Use Committee at Albany Medical College reviewed and approved the animal protocols (ACUP #24–03003, 24–04003) in accordance with the Association for Assessment and Accreditation of Laboratory Animal Care, the US Department of Agriculture, and the US Public Health Service guidelines. Euthanasia of animals was performed in accordance with the American Veterinary Medical Association (AVMA) guidelines. This study adheres to the ARRIVE guidelines for reporting animal studies.

### Mice

8–10-week-old C57BL/6 (Strain #:000664), *Il1r1*^*−/−*^ (Strain #:003245), and C3HeB/FeJ (Strain #:000658) mice were purchased from The Jackson Laboratory. *Fpr1*^*−/−*^ and *Fpr2*^*−/−*^ mice were kindly donated by Dr Ji Ming-Wang of the National Cancer Institute, at The National Institutes of Health, Bethesda, MD. Animals were bred and maintained under Specific Pathogen-Free conditions at Albany Medical College. All mouse studies were conducted in accordance with protocols approved by the AMC Institutional Animal Care and Use Committee (IACUC) (Animal Care User Protocol Number ACUP-24–03003, 24–04003). Care was taken to minimize pain and suffering in Mtb-infected mice.

### Mouse infections

A single-cell suspension of Mtb HN878 smyc’::mCherry strains was prepared in Phosphate Buffered Saline (PBS) containing 0.05% Tween 80 (PBST). To disperse clumps, the suspension was passed through 18- and 21-gauge needles, respectively. Approximately 100 colony-forming units (CFU) of bacteria were used for aerosol route infection employing an aerosol-generating device (Glas-Col inhalation exposure system, Terre Haute, IN) as described previously^50,51^. The evaluation of infection was carried out by enumerating bacterial CFUs in lung and spleen homogenates from infected mice at Day 29 post-infection, using serial dilutions and plating on 7H10 Agar plates enriched with 0.5% v/v Glycerol and Middlebrook OADC enrichment. Colony counting was performed on plates after three weeks of incubation at 37°C.

### Bacterial strains

Throughout this study, the hypervirulent *Mycobacterium tuberculosis* HN878 strain was utilized. Strains of Mtb HN878 were genetically modified with fluorescence reporters, including smyc’::mCherry, while maintaining resistance to Hygromycin B. The bacteria were cultured in Middlebrook 7H9 media (Becton Dickinson) supplemented with OADC (Becton Dickinson), 0.05% Tween 20, 0.5% v/v Glycerol, and 50 μg/ml Hygromycin B in a shaking incubator at 37°C for 5–7 days until they reached the log phase growth. The strains were preserved at −80°C in 20% glycerol until further use for infection studies.

### RNA isolation and Real-Time PCR

For gene expression studies, cells were isolated from the lungs at 27 days post-infection from both Wt and *Il1r1*^*−/−*^ mice. The mRNA from the lung cells was extracted using the RNeasy Mini kit (Cat.: 74104, QIAGEN), as instructed by the manufacturer. The concentration and purity of RNA samples were determined by spectroscopy at 260/280 nm and 260/230 nm, respectively. RNA integrity was analyzed through electrophoresis using a 1% agarose gel. cDNA synthesis was carried out using the SuperScript^™^III two-step RT-PCR System with Platinum^™^ Taq DNA Polymerase, reagents, and protocol provided by the manufacturer (ThermoFisher Scientific, USA). The primers were designed using Integrated DNA Technologies PrimerQuest software (www.idtdna.com/site). Ubiquitin was used as the housekeeping gene. The cDNA was subjected to SYBR Green RT-PCR assay using primers and Luna^®^ Universal qPCR Master Mix (Biolabs, USA) in the StepOnePlus RT-PCR system (Applied Biosystems, USA) at 95°C for 60 seconds, followed by 40 cycles consisting of denaturation at 95°C for 15 secs, annealing at 55°C for 10 secs, and extension at 60°C for 30 secs. Following amplification, determination of threshold cycle (CT) values and melting curve analysis were carried out. The analysis was carried out following the MIQE guidelines for real-time PCR experiments.

### Immunofluorescence Microscopy:

Lung lobes were fixed overnight in 10% buffered formalin and embedded in paraffin. Tissue sections were cut at 5 μm thickness and mounted on ultraclean glass slides. Paraffin-embedded lung tissue sections were processed according to the method described by Abcam. In brief, tissue sections were deparaffinized and rehydrated by: xylene for 3 mins (2 times), xylene + 100% ethanol (1:1) for 3 mins, 100% ethanol for 3 mins (2 times) followed by 95%, 70%, and 50% ethanol for 3 mins each, respectively. Finally, slides were kept in distilled water for 20 mins. Heat-induced epitope retrieval method was utilized to perform antigen retrieval by boiling slides in sodium citrate buffer (pH 6.0) for 20 mins. After cooling down the slides, the section was subjected to permeabilization by dipping in PBS containing 0.2% Triton X-100 (Sigma-Aldrich) and 0.05% Tween 20 (Sigma-Aldrich) for 10 mins. Slides were incubated with 5% BSA for 2 hours at room temperature to avoid nonspecific binding. After washing slides with wash buffer (PBS containing 0.05% Tween 20), slides were incubated with primary antibodies overnight at 4°C. Primary antibodies used were: anti-FPR1 antibody (Cat: FPR1–101AP, Fabgennix), and anti-FPRL1/FPR2 antibody (Cat: NLS1878SS, Novus Biologicals). Following incubation with the primary antibody, slides were incubated with the respective secondary antibodies (anti-rabbit conjugated 667, Cat: ab6564, Abcam) for at least 2 hours at room temperature. Tissues were washed and mounted using Prolong Gold Antifade reagent (Invitrogen, Grand Island, NY) with DAPI. Tissue sections were examined using an ECHO Revolve 4 microscope. Images were analyzed using image J software.

### Flow cytometry

Lungs were collected in ice cold PBS from Mtb-infected mice at designated time points. To obtain single cell suspension, lung tissues were digested with Collagenase type IV (150 U/mL) (Cat 17104019, Gibco) and DNase I (60 U/mL) (Cat: 10104159001, Roche-Sigma Aldrich) cocktail. After digestion, the suspension was filtered through 40 μm cell strainers. The cell suspension was subjected to red blood cell lysis by using ACK lysis buffer (Cat: BP10–548E, Lonza) to obtain single-cell suspensions for further staining. Non-specific binding was prevented by incubating the single-cell suspension with Fc-Block CD16/32 in FACS buffer (PBS + 0.5% BSA) (Cat: 156604, BioLegend). Surface staining was performed by staining cells in the dark with directly fluorescently conjugated antibodies for 30 mins at 4°C in FACS buffer. Cells were fixed with Fixation Buffer (Cat: 420801, BioLegend) according to the manufacturer’s instructions. Samples were acquired on a BD Symphony^™^ flow cytometer, and all analyses were done in FlowJo v10. All analyses were conducted on viable cells. The exclusion of dead cells was achieved using the fixable viability stain conjugated with eFluor780 (Cat: 65-0864-14, eBioscience). Further gating to analyze various populations was as follows: neutrophils: CD11b^+^Ly6G^+^, macrophages: CD11b^+^Ly6G^−^ CD11c^+^MHCII^+^SiglecF^−^, Monocytes: CD11b^+^Ly6G^−^CD11c^−^, B cells: CD19^+^, CD4^+^ T cells: CD19^−^CD8^−^CD4^+^, CD8^+^ T cells: CD19^−^CD8^+^CD4^−^. The antibodies used to analyze myeloid cells included: CD11b (Clone M170), Ly6G (Clone 1A8), Ly6C (Clone HK1.4), I-A/I-E (Clone M5/114), Siglec F (Clone 1RMM44N), CD11c (Clone N418). Antibodies used to analyze lymphoid cells included: CD4 (Clone GK 1.5), CD8 (Clone 53–6.7), CD19 (Clone 6D5). All antibodies were purchased from the BioLegend inc.

### Histopathology

Lung lobes were fixed overnight in 10% buffered formalin and embedded in paraffin. Hematoxylin and eosin (H&E) staining was done on 5 um thick lung sections by histopathology core facility at the Albany Medical College. NanoZoomer 2.0 RS Hamamatsu slide scanner was used to image H&E-stained slides. All quantification was done by blind scoring method using image J software.

### Neutrophil purification and ex-vivo infection

Bones from naïve C57BL/6, *Fpr1*^*−/−*^, *Pad4*^*−/−*^, *Cybb*^*−/−*^ mice were flushed with DMEM media containing Sodium Pyruvate, Sodium Bicarbonate, HEPES, and 10% FBS. Flushed cells from bone marrow were passed through 18-gauge needles to disrupt clumps. Red blood cells were lysed using ACK lysis buffer (Cat: BP10–548E, Lonza) to obtain single-cell suspensions. Neutrophils were isolated by magnetic sorter using the Mojo sort neutrophil isolation kit (Biolegend Cat: 480058) as suggested by manufacturer. In brief, single-cell suspensions from bone marrow were washed using Mojo sort buffer (Cat: 480017, BioLegend). After washing, cells were incubated with the biotinylated antibody cocktail (1:10 in Mojo sort buffer) for 30 minutes. Cells were then subjected to incubation with bead-bound secondary streptavidin for 30 minutes (1:10 in Mojo sort buffer). Finally, washed cells were incubated for 5 minutes over a magnet (Cat: 480019, BioLegend). Purified neutrophils were collected by negative sorting, collecting unbound cells. The purity of collected neutrophils was checked by flow cytometry using CD11b (Clone M170) and Ly6G (Clone 1A8) surface staining. Mtb HN878 smyc’::mCherry single-cell suspension was prepared as mentioned earlier. Purified neutrophils were infected at a 3 Multiplicity of Infection (MOI).. After infection, neutrophils were incubated at 37°C with 5% CO_2_ for 24 hours. At 4 hours post-infection (p.i.), cells were washed with completely fresh culture media to remove extracellular bacteria. At 24 hours p.i., cells were collected for CFU analysis.

### Bone Marrow derived Macrophage (BMDM) generation and ex-vivo infection

Bones from naïve C57BL/6 and *Fpr1*^−/−^mice were flushed, and cell suspensions were prepared as mentioned above. The ACK-lysed cell suspension was cultured for 5–7 days in DMEM media containing L929-conditioned media, Sodium Pyruvate, Sodium Bicarbonate, HEPES, and 10% FBS. Differentiated BMDMs were infected with a MOI=3.0 of bacteria. At 4 hours post-infection (p.i.), cells were washed with completely fresh culture media to remove extracellular bacteria. Cells were collected on day 3, 5, and 7 days p.i. for CFU analysis.

### Bacterial burden enumeration by CFU analysis

Infected neutrophils and BMDMs were collected at their respective time points. Cells were lysed with PBS + 0.1% Triton X100 for 5 minutes at room temperature. Serially diluted bacteria were plated on 7H10 agar plates with 0.5% v/v Glycerol and OADC enrichment. Plates were incubated at 37°C for 3 weeks. To enumerate bacterial burden from in vivo experiments, lung lobes and spleens from infected mice were homogenized using Matrix lysing tubes (Cat: 116913500, MP Bio) containing PBST. Using a bead beater homogenizer (BioSpec, Mini-Bead beater), tissues were homogenized for one minute with a-minute interval for three times. After homogenization, samples were serially diluted and plated for bacterial colony-forming units as mentioned above.

### FPR1 inhibition

FPR1 inhibitors were administered to infected *Il1r1*^*−/−*^ and C3HeB/FeJ mice via an oral gavage route. FPR1 inhibitors: Cyclosporin H (4mg/kg,) (Cat: HY-P1122, MCE) and HCH6–1 (4mg/kg) (CatHY-101283, MCE). Treatment started day 1 infection and continued every other day till day 25 post infection for *Il1r1*^*−/−*^ or 35 post infection for C3HeB/FeJ mice.

### Statistics

Statistical differences among the specified groups were assessed using unpaired two-tailed Student’s t-tests or two-way Analysis of Variance (ANOVA) with Tukey’s multiple comparison tests. All statistical analyses were conducted with Graph Pad Prism 10 software. A significance level of p <0.05 was considered statistically significant. The figures and figure legends indicate the values of ‘n’ as well as other relevant statistical values (*: p < 0.05; **: p < 0.01; ***: p < 0.001).

## Data Availability

All the data and resources generated during this study are available upon request to the corresponding author.
